# Vibrio cholerae Isolation from Frozen Vomitus and Stool Samples

**DOI:** 10.1128/jcm.01084-22

**Published:** 2022-09-28

**Authors:** Chelsea N. Dunmire, Denise Chac, Fahima Chowdhury, Ashraful I. Khan, Taufiqur R. Bhuiyan, Regina C. LaRocque, Afroza Akter, Mohammad Ashraful Amin, Edward T. Ryan, Firdausi Qadri, Ana A. Weil

**Affiliations:** a Department of Medicine, University of Washington, Seattle, Washington, USA; b Infectious Diseases Division, International Center for Diarrheal Disease Research, Bangladesh (icddr,b), Dhaka, Bangladesh; c Massachusetts General Hospital, Boston, Massachusetts, USA; Medical College of Wisconsin

**Keywords:** isolation, *Vibrio cholerae*

## LETTER

The gold standard for diagnosing cholera is culture of fresh stool enriched in alkaline peptone water (APW) for 6 to 8 h with plating onto thiosulphate citrate bile salts (TCBS) agar, where Vibrio cholerae (*Vc*) appears as bright yellow colonies (1–3). Optimal methods for *Vc* recovery from frozen clinical samples have not been established (4).

We tested *Vc* recovery in stool and vomitus from patients with acute, severe cholera. Samples were collected from patients presenting to the International Center for Diarrheal Diseases Research, Bangladesh, prior to antibiotic treatment. A total of 50 mL vomitus and 50 mL stool from each participant was stored in 30% glycerol, and another 50 mL of each sample was stored without glycerol. Samples were immediately frozen at −80°C. Routine diagnostics were performed using fresh samples, including CFU counts of presumed *Vc* from samples plated on taurocholate-tellurite-gelatin agar (5). Among 20 individuals’ stool and vomitus samples, the mean recovery of *Vc* CFU/mL from fresh samples was 1.8 × 10^7^ and 5.4 × 10^6^, respectively. After shipping on dry ice and storage at −80°C for 1 year, samples were thawed and 100 μL was inoculated into Luria-Bertani broth (LB, Difco, Franklin Lakes, NJ) and APW (Oxoid, Basingstoke, United Kingdom) for enrichment, with subsequent plating onto LB and TCBS agar (Table 1). Next, 30 μL of each sample was also streaked directly onto LB, TCBS, and tryptic soy agar with 5% sheep’s blood (TSAb; Remel, Lenexa, KS). Direct plated and enrichment broths were incubated overnight at 37°C, and the next day, enrichment broths were plated onto agars listed in Table 1 for repeat overnight culture (2). *Vc* colonies isolated were confirmed by PCR amplification of cholera toxin subunit A and O1/O139 rfb regions (6). pH of frozen unpreserved samples was measured using a Sartorius meter (Gottingen, Germany), to determine if pH impacted ability to recover *Vc*. The icddr,b, Massachusetts General Hospital, and University of Washington approved this study.

**TABLE 1:** Media used to isolate Vibrio cholerae from frozen, stored rice-water stool and vomitus from cholera patients^*a*^

	VOMITUS													
	No preservative							Cultured from + glycerol						
Patient	LB	TSAb	TCBS	LB > LB	APW > LB	LB > TCBS	APW > TCBS	LB	TSAb	TCBS	LB > LB	APW > LB	LB > TCBS	APW > TCBS
1	X	X	−	X	X	X	X	ND	ND	ND	ND	ND	ND	ND
2	−	−	−	−	−	−	−	−	−	−	−	X	−	X
3	−	−	−	−	−	−	−	−	−	−	−	−	−	X
4	−	−	−	−	−	−	−	X	X	−	−	X	−	X
5	−	−	−	−	−	−	−	X	X	−	−	X	−	X
6	−	X	−	X	X	−	−	ND	ND	ND	ND	ND	ND	ND
7	−	−	−	−	−	−	X	−	−	−	−	−	−	−
8	−	−	−	−	−	−	−	−	X	−	−	−	−	−
9	−	−	−	−	−	−	−	X	X	−	−	X	−	X
10	−	−	−	−	−	−	−	X	X	−	−	X	−	X
11	−	−	−	X	−	X	−	ND	ND	ND	ND	ND	ND	ND
12	−	−	−	X	X	−	X	−	−	−	−	−	−	−
	**STOOL**													
1	X	X	−	X	X	X	X	ND	ND	ND	ND	ND	ND	ND
2	X	X	−	X	X	X	X	ND	ND	ND	ND	ND	ND	ND
3	X	X	−	X	X	−	−	ND	ND	ND	ND	ND	ND	ND
4	−	−	−	X	X	X	X	ND	ND	ND	ND	ND	ND	ND
5	−	−	−	X	X	X	X	ND	ND	ND	ND	ND	ND	ND
6	−	−	−	X	X	−	−	ND	ND	ND	ND	ND	ND	ND
7	−	−	−	X	−	−	X	ND	ND	ND	ND	ND	ND	ND
8	−	−	−	−	−	−	−	X	X	−	X	X	−	X
9	−	−	−	−	−	−	−	X	X	−	−	X	−	−
10	−	−	−	−	−	−	−	X	X	−	−	−	−	−
11	−	−	−	−	−	−	−	X	−	−	−	−	−	−
12	−	−	−	−	−	−	−	−	X	−	X	−	X	−
13	−	−	−	−	−	−	−	−	−	−	−	−	X	−

a X represents successful isolation (the culture grew *V. cholerae*), “−” indicates a negative culture (*V. cholerae* did not grow). “>” denotes enrichment in broth (first set of initials) for 24 h prior to plating on agar (second set of initials). All direct plated samples were incubated at 37°C overnight, and enrichment samples underwent one overnight incubation in enrichment media and another after plating onto agar. Overnight enrichment was chosen because this interval was previously found to be equivalent to 6 to 8 h enrichment for *Vc* recovery (2). If samples stored without preservative did not yield *Vc*, glycerol-preserved samples were cultured. APW incubations were conducted in nonshaking culture, and LB incubations were in shaking culture at 225 rpm. Patient samples with no successful *Vc* isolations (7 stool samples and 8 vomitus samples) are not shown. ND, not done; LB, Luria-Bertani; TSAb, tryptic soy agar with 5% sheep’s blood; TCBS, thiosulphate citrate bile salts agar; APW, alkaline peptone water.

**FIG 1:**
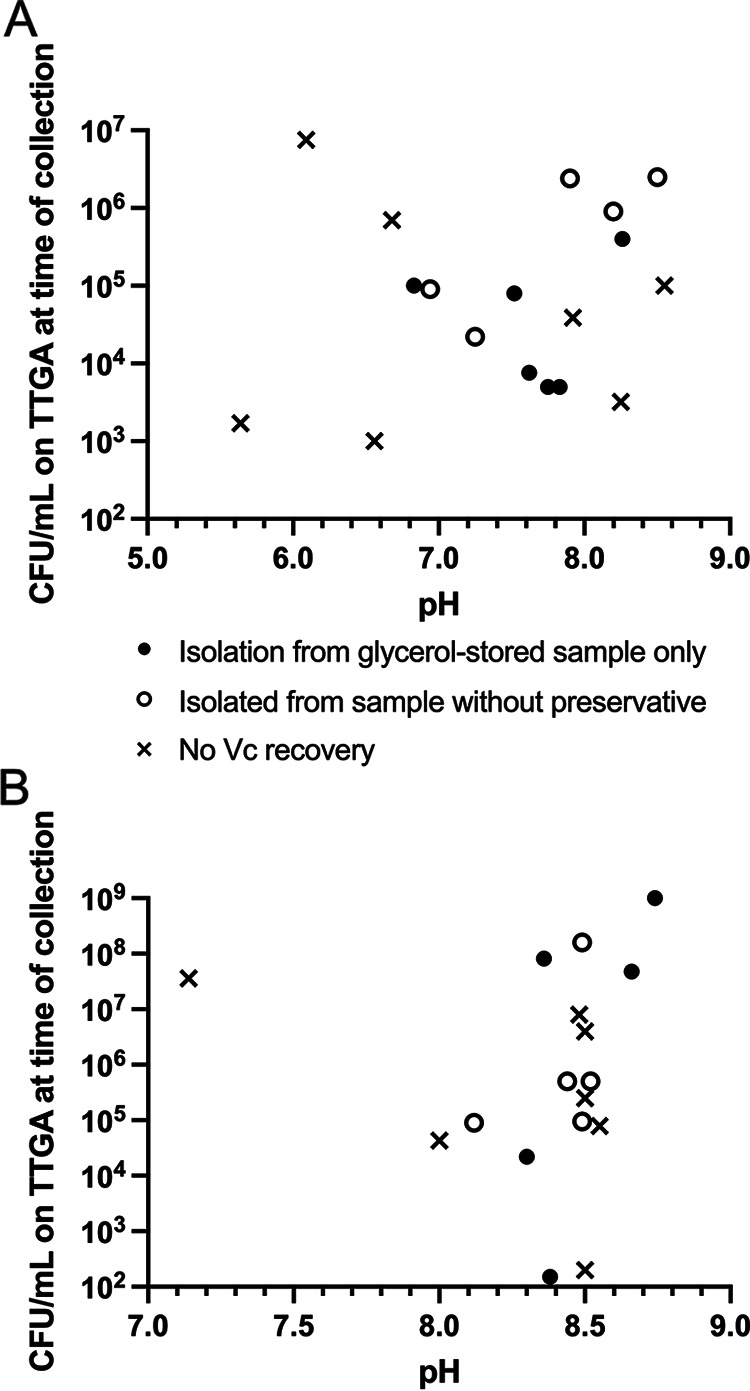
Vibrio cholerae (*Vc*) isolation and pH from a subset of frozen samples and the relationship between these factors and CFU count from the fresh sample; *n* = 18 for each sample type. *Vc* CFU were enumerated from fresh plating of stool selective tellurite taurocholate gelatin agar. (A) *Vc* isolation of vomitus and (B) stool samples. Each data point represents one sample. “Isolated from sample without preservative” indicates that *Vc* was isolated from samples stored in no preservative. If this method of isolation failed, samples stored in glycerol were attempted, and successful where “Isolation from glycerol-stored sample only” is indicated. If both glycerol-preserved samples and no preservative samples failed *Vc* isolation, “No *Vc* recovery” is shown. Three of 20 participants had one or both samples omitted from this figure: One study participant’s vomitus and stool samples are not included because only a glycerol culture for *Vc* was performed. One study participant’s fresh vomitus sample had no CFU on TTGA culture (and stool was TTGA *Vc* positive); thus, the vomitus sample was omitted from this figure. Another single study participant’s fresh stool sample had no CFU on TTGA culture (and vomitus was TTGA *Vc* positive); thus, the stool sample was omitted from this figure. One stool CFU count was “uncountable,” shown on this figure at 10^9^.

*Vc* isolation was successful in 13 of 20 frozen stool and 12 of 20 frozen vomitus samples (25/40, 63%). For patients in which isolation was successful, *Vc* yield from direct plating was comparable to gold standard enrichment methods. Direct plating of stool (LB or TSAb) was successful in 8 of 13 stool samples and 9 of 13 using APW enrichment (*X^2^* value 1.7, *P* = 0.68). Isolation was successful in 7 of 12 vomitus samples with direct plating, and 9 of 12 with APW enrichment (*X^2^* value 0.8, *P* = 0.39). *Vc* recovery was independent of pH or CFU counts from the fresh sample (Fig. 1) (7). Potential reasons for failure of *Vc* recovery include freezing-related killing and the possibility that *Vc* enters a viable but nonculturable state when frozen for long time periods (8). In summary, we found that direct plating of stool and vomitus onto LB or TSAb agar was a successful method for *Vc* isolation from frozen clinical samples (stool and vomitus) and may reduce supplies and labor needed for *Vc* isolation. While direct plating of samples onto nonselective media such as LB or TSAb allows growth of other bacteria, we found that the morphology of *Vc* colonies were easily recognizable in these frozen samples from acute illness.

## References

[1] World Health Organization. 1987. Manual for the laboratory investigations of acute enteric infections. Program for Control of Diarrheal Diseases, World Health Organization, Geneva, Switzerland.

[2] Lesmana M, Richie E, Subekti D, Simanjuntak C, Walz SE. 1997. Comparison of direct-plating and enrichment methods for isolation of *Vibrio cholerae* from diarrhea patients. J Clin Microbiol 35:1856–1858. doi:10.1128/jcm.35.7.1856-1858.1997. 9196208 PMC229856

[3] Rennels MB, Levine MM, Daya V, Angle P, Young C. 1980. Selective vs, nonselective media and direct plating vs. enrichment technique in Isolation of *Vibrio cholerae*: recommendations for clinical laboratories. J Infect Dis 142:328–331. doi:10.1093/infdis/142.3.328. 7003031

[4] Dan M, Richardson J, Miliotis MD, Koornhof HJ. 1989. Comparison of preservation media and freezing conditions for storage of specimens of faeces. J Med Microbiol 28:151–154. doi:10.1099/00222615-28-2-151. 2915366

[5] Khan AI, Rashid MM, Islam MT, Afrad MH, Salimuzzaman M, Hegde ST, Zion MMI, Khan AH, Shirin T, Habib ZH, Khan IA, Begum YA, Azman AS, Rahman M, Clemens JD, Flora MS, Qadri F. 2020. Epidemiology of cholera in Bangladesh: findings from nationwide hospital-based surveillance, 2014–2018. Clin Infect Dis 71:1635–1642. doi:10.1093/cid/ciz1075. 31891368

[6] Hoshino K, Yamasaki S, Mukhopadhyay AK, Chakraborty S, Basu A, Bhattacharya SK, Nair GB, Shimada T, Takeda Y. 1998. Development and evaluation of a multiplex PCR assay for rapid detection of toxigenic *Vibrio cholerae* O1 and O139. FEMS Immunol Med Microbiol 20:201–207. doi:10.1111/j.1574-695X.1998.tb01128.x. 9566491

[7] Huq A, West PA, Small EB, Huq MI, Colwell RR. 1984. Influence of water temperature, salinity, and pH on survival and growth of toxigenic *Vibrio cholerae* serovar 01 associated with live copepods in laboratory microcosms. Appl Environ Microbiol 48:420–424. doi:10.1128/aem.48.2.420-424.1984. 6486784 PMC241529

[8] Colwell RR, Huq A. 1994. *Vibrios* in the environment: viable but nonculturable *Vibrio cholerae* p 117–133. *In* Wachsmuth K, Blake PA, Olsvik O (ed), Vibrio cholerae and cholera: molecular to global perspectives. American Society for Microbiology. Washington, DC.

